# Can rodent models elucidate the pathomechanisms of genetic epilepsy?

**DOI:** 10.1111/bph.15443

**Published:** 2021-05-12

**Authors:** Motohiro Okada

**Affiliations:** ^1^ Department of Neuropsychiatry, Division of Neuroscience, Graduate School of Medicine Mie University Tsu Japan

**Keywords:** acetylcholine, connexin, genetic epilepsy, glutamate, hemichannel, pathomechanism

## Abstract

Autosomal dominant sleep‐related hypermotor epilepsy (ADSHE; previously autosomal dominant nocturnal frontal lobe epilepsy, ADNFLE), originally reported in 1994, was the first distinct genetic epilepsy shown to be caused by *CHNRA4* mutation. In the past two decades, we have identified several functional abnormalities of mutant ion channels and their associated transmissions using several experiments involving single‐cell and genetic animal (rodent) models. Currently, epileptologists understand that functional abnormalities underlying epileptogenesis/ictogenesis in humans and rodents are more complicated than previously believed and that the function of mutant molecules alone cannot contribute to the development of epileptogenesis/ictogenesis but play important roles in the development of epileptogenesis/ictogenesis through formation of abnormalities in various other transmission systems before epilepsy onset. Based on our recent findings using genetic rat ADSHE models, harbouring *Chrna4* mutant, corresponding to human S284L‐mutant *CRHNA4*, this review proposes a hypothesis associated with tripartite synaptic transmission in ADSHE pathomechanisms induced by mutant ACh receptors.

**LINKED ARTICLES:**

This article is part of a themed issue on Building Bridges in Neuropharmacology. To view the other articles in this section visit http://onlinelibrary.wiley.com/doi/10.1111/bph.v179.8/issuetoc

**Abbreviations**
ADNFLE: autosomal dominant nocturnal frontal lobe epilepsy
ADSHE: autosomal dominant sleep‐related hypermotor epilepsy
DAC: dystonic‐arousal complex
EEG: electroencephalogram
ENW: episodic nocturnal wandering
EPSC: excitatory synaptic current
IPSC: inhibitory postsynaptic current
KCC: potassium chloride cotransporter
M2C: secondary motor cortex
mCx43: connexin43 in the plasma membrane
MDTN: mediodorsal thalamic nucleus
MoTN: motor thalamic nuclei
NKCC: sodium–potassium chloride cotransporter
NPA: nocturnal paroxysmal arousals
NPD: nocturnal paroxysmal dystonia
OFC: orbitofrontal cortex
RTN: reticular thalamic nucleus
SHE: sleep‐related hypermotor epilepsy syndrome
STN: subthalamic nucleus
VDSC: voltage‐dependent Na^+^ channel

## 1 INTRODUCTION: AUTOSOMAL DOMINANT SLEEP‐RELATED HYPERMOTOR EPILEPSY

Autosomal dominant sleep‐related hypermotor epilepsy (ADSHE: previously known as autosomal dominant nocturnal frontal lobe epilepsy [ADNFLE]) is a subfamily of sleep‐related hypermotor epilepsy syndrome (SHE), the first genetic epilepsy identified to be caused by a mutation in *CHNRA4* , the gene for the α4 subunit of the nAChR, as described in 1994 (Scheffer et al., 1994; Tinuper et al., 2016). Numerous mutations associated with ADSHE in various genes, including *CRHNA4* (Cho et al., 2003; Hirose et al., 2000; Ito et al., 2000; Magnusson et al., 2003; McLellan et al., 2003; Miyajima et al., 2013; Phillips et al., 2000; Rozycka et al., 2003; Saenz et al., 1999; Sansoni et al., 2012; Steinlein et al., 1995, 1997, 2000), *CRHNB2* (Bertrand et al., 2005; Cho et al., 2008; De Fusco et al., 2000; Diaz‐Otero et al., 2008; Gambardella et al., 2000; Hoda et al., 2008; Leniger et al., 2003; Phillips et al., 2001), *CRH* (Combi et al., 2005, 2008; Sansoni et al., 2013), *KCNT1* (Heron et al., 2012), *CABP4* (Chen et al., 2017), *DEPDC5* (Ishida et al., 2013) and *GATOR1* (Baldassari et al., 2016), have been identified from the ADSHE pedigrees. Recent results have shown that ADSHE can be caused by genes not encoding ion channels or not directly related to the cholinergic system, such as *CRH, DEPDC5* and *GATOR1* (Baldassari et al., 2016; Combi et al., 2005; Ishida et al., 2013). In particular, mutations associated with *CRH* in its promoter regions have also been identified (Combi et al., 2005), suggesting that abnormalities in the expression level of target proteins might be involved in the pathomechanisms of ADSHE, unlike other mutations.

The functional abnormalities of ADSHE‐mutant nicotinic acetylcholine receptor (nAChR) were determined in transfected single‐cell, *Xenopus* oocytes or human embryonic kidney (HEK) cells using the whole‐cell patch‐clamp technique as a rapid functional screening device, because a number of epileptologists required evidence that the mutant genes identified in ADSHE pedigrees did actually affect ADSHE pathomechanisms. As expected, single‐cell models indicated various functional abnormalities of ADNFLE‐mutant nAChRs, which could suggest several candidate pathomechanisms of ADNFLE (Bertrand et al., 2002; Figl et al., 1998; Picard et al., 1999; Rodrigues‐Pinguet et al., 2003, 2005). Subsequently, epileptologists also demanded certification of whether experimental animal models harbouring mutations corresponding to human mutation could manifest the epileptic symptoms of patients with ADSHE. To respond to these demands, various genetic rodent ADNFLE models have been generated using knock‐in (KI) or transgenic (TG) technologies. S280F‐mutant *CRHNA4* models (previously designated “S248F” according to reference sequence NP_000735.1) were generated in two mouse strains and named S252F‐KI and S248F‐KI (Klaassen et al., 2006; Teper et al., 2007). The insL‐mutant *CRHNA4* model was generated in one mouse strain, named insL‐KI (Klaassen et al., 2006). S284L‐mutant models (previously designated “S252L” according to the reference sequence NP_000735.1) generated two rat strains, named S284L‐TG and S286L‐TG (K. Fukuyama, Fukuzawa, & Okada, 2020; K. Fukuyama, Fukuzawa, Okubo et al., 2020; Fukuyama et al., 2020a, 2020b; Fukuyama & Okada, 2020; Yamamura et al., 2013; Zhu et al., 2008). V287L‐mutant *CRHNB2* models were generated in two mice strains, named V287L‐KI and V287L‐TG, and one rat strain, named V286L‐TG (Gullo et al., 2014; Manfredi et al., 2009; O'neill et al., 2013; Shiba et al., 2015; Xu et al., 2011).

Over the past two decades, such experiments using single‐cell and KI mice models of ADSHE have provided some elucidation of ADSHE pathogenesis, though even more contradictions have subsequently emerged. However, exploring the functional abnormalities of the typical ADSHE‐mutant α4 and β2 subunits of nAChR has remained a significant target in understanding ADSHE pathomechanisms (Becchetti et al., 2015; Boillot & Baulac, 2016). Recently, a part of the outline of ADSHE pathomechanisms was clarified using a transgenic rat model bearing S286L‐mutant rat *Chrna4*, corresponding to the S284L‐mutation in human *CRHNA4* of patients with ADSHE. However, the identified functional abnormalities associated with ADSHE were also more complicated than expected. Complex pathomechanism cascades of ADSHE originate from functional abnormalities of mutant α4β2‐nAChR, from alterations of its cation channel to wide‐range abnormalities, involving unexpected intracellular signalling (K. Fukuyama, Fukuzawa, Okubo et al., 2020; Fukuyama et al., 2020b; Fukuyama & Okada, 2020; Fukuyama, Ueda et al., 2020). These experimental findings, which reveal various functional abnormalities originating from mutant α4β2‐nAChR, also elucidated the pathogenesis of the age‐dependent onset and clinical features of the ADSHE subclass, including comorbid cognitive impairment and anticonvulsant sensitivity (K. Fukuyama, Fukuzawa, Okubo et al., 2020; Fukuyama et al., 2020b; Fukuyama & Okada, 2020; Fukuyama, Ueda et al., 2020; Picard et al., 1999; Yamamura et al., 2013; Zhu et al., 2008). Full elucidation of the complex pathophysiological cascade associated with ADSHE will contribute to uncovering the mechanisms of genetic epilepsies caused by other gene mutations. Furthermore, it may lead to breakthroughs in understanding the common pathomechanisms of focal epilepsies. Therefore, this review discusses the pathomechanisms of ADSHE, focusing on S284L‐mutant ADSHE, comparing the functional abnormalities using single‐cell and KI mouse models bearing other classical ADSHE mutations, such as S280F, insL and V287L.

## 2 CLINICAL FEATURES OF ADSHE/SHE SYNDROME

The clinical manifestations of ADSHE and sporadic SHE are indistinguishable, because they are comparable to frontal lobe epilepsy and usually occur during non‐rapid eye movement (non‐REM) sleep (Provini et al., 1999; Scheffer et al., 1995; Tinuper et al., 2016). SHE is a rare form of focal epilepsy, with an estimated prevalence of 1.8/100,000, thus fulfilling the definition of a rare disease (Tinuper et al., 2016). ADSHE seizures are sleep‐related, stereotyped hypermotor seizures consisting of vigorous hyperkinetic features or asymmetric dystonic/tonic features. In addition, many paroxysmal arousals are observed. More rarely in some patients, seizures can also manifest as epileptic nocturnal wandering. Patients usually experience a cluster of hypermotor seizures during the same night. Electroencephalogram (EEG) often fails to detect ictal discharge during hypermotor seizures (Tinuper et al., 2016). These brief seizures may sometimes evolve into secondary generalised tonic–clonic seizures (Tinuper et al., 2016). Even if ADSHE seizures are controlled, once a patient experiences a seizure, they can experience clustering and many/frequent ADSHE seizures during the same night (Provini et al., 1999; Scheffer et al., 1995). Based on these clinical findings, it has been deemed that ADSHE seizures (sleep‐related complex, stereotyped hypermotor seizures) probably represent the spectrum of SHE syndrome, despite differences in EEG sensitivities (Montagna, 1992; Provini et al., 1999; Tinuper et al., 2016).

Traditionally, ADNFLE seizures were classified into three typical types: nocturnal paroxysmal arousals (NPA), nocturnal paroxysmal dystonia (NPD) and episodic nocturnal wandering (ENW) (Provini et al., 1999). Clinically, the terminologies describing NPA, NPD and ENW are currently outdated in patients with ADSHE. However, these three typical ADNFLE seizures are the major clinical terminologies, at the generation of genetic ADNFLE rodent models. Therefore, in this review, the epileptic phenotypes of genetic ADNFLE rodent models were defined by NPA, NPD and ENW.

ADSHE/SHE are classified into two subclasses based on anticonvulsant sensitivity and cognitive dysfunction (Picard et al., 1999; Tinuper et al., 2016). Genetic variations appear to be closely associated with the clinical features of subclass formation (Table 1). Approximately 60% of SHE patients, including ADSHE subjects with S280F (Steinlein et al., 1995, 2000) and insL mutations of *CRHNA4* (Steinlein et al., 1997) and V287L‐mutant *CRHNB2* (Gambardella et al., 2000), can achieve remission and exhibit improved prognosis with relatively low doses of carbamazepine (Picard et al., 1999; Provini et al., 1999). In contrast, over 30% of SHE patients, including those with the *CRHNA4* S284L mutation (Combi et al., 2004; Hirose et al., 1999; Ito et al., 2000; Miyajima et al., 2013), gain no benefits from carbamazepine therapy (Provini et al., 1999). ADSHE seizures are usually the sole, primary neurological symptom of this syndrome, with less than 3% of subjects presenting with other psychiatric disturbances (Gambardella et al., 2000; Miyajima et al., 2013; Provini et al., 1999; Steinlein et al., 1995, 2000). In contrast, ADSHE with the insL and S284L mutations present comorbid cognitive impairments, including schizophrenia‐like psychosis, autism and intellectual disability (Cho et al., 2003; Ito et al., 2000; Magnusson et al., 2003; Miyajima et al., 2013; Phillips et al., 2000; Steinlein et al., 1997).

**TABLE 1:** Clinical manifestation of ADSHE‐mutant nAChRs

Gene	CRHNA4		CRHNB2	
Mutation (aminoacid change)	c.839C > T (S280F)	c.870_872dupGCT (L291dup: insL)	c.851C > T (S284L)	c.859G > C (V287L)
Comorbidity	No neuropsychological disturbance	Psychosis	Intellectual disabilities autism	No neuropsychological disturbance
CBZ susceptibility	Good	Good	Poor	Good
References	[1–4]	[5, 6]	[7–13]	[14, 15]

References: 1. Cho et al. (2003); 2. De Fusco et al. (2000); 3. Gambardella et al. (2000); 4. Hirose et al. (2000); 5. Ito et al. (2000); 6. Magnusson et al. (2003); 7. McLellan et al. (2003); 8. Miyajima et al. (2013); 9. Phillips et al. (2000); 10. Rozycka et al. (2003); 11. Saenz et al. (1999); 12. Sansoni et al. (2012); 13. Steinlein et al. (1997); 14. Steinlein et al. (1995); 15. Steinlein et al. (2000).

## 3 FUNCTIONAL ABNORMALITY OF MUTANT SUBUNITS EXPRESSED IN SINGLE‐CELL MODELS

Initial studies found that both S280F‐ and insL‐mutant α4β2‐nAChRs had enhanced sensitivity to ACh and displayed use‐dependent potentiation (Table 2) (Bertrand et al., 1998, 2002; Figl et al., 1998; Kuryatov et al., 1997; Moulard et al., 2001; Weiland et al., 1996). The similarity in functional abnormalities between S280F‐ and insL‐mutant α4β2‐nAChRs led to initial optimism among researchers to elucidate ADSHE pathogenesis, because the combination of enhanced ACh sensitivity and use‐dependent potentiation was a simple mechanism based on the imbalance hypothesis (Hirose et al., 2000). Clinical studies in patients with ADSHE have revealed the upregulation of α4β2‐nAChRs in the mesencephalon, projecting through cholinergic pathway (brainstem ascending cholinergic system) to the thalamus (Picard et al., 2006). Additionally, ADNFLE seizures mainly occur during Stage 2 of non‐REM sleep and often appear to arise from sleep spindles that originate in the thalamus (Ito et al., 2000; Picard et al., 2006; Provini et al., 1999; Tinuper et al., 2016). Thus, taken together with clinical findings, it was initially considered that the ACh release induced by sleep spindle or pathological interictal/ictal discharges, can activate mutant α4β2‐nAChR at relatively lower ACh concentrations compared to wild‐type α4β2‐nAChRs.

**TABLE 2:** Functional abnormalities of ADSHE‐mutant nAChRs

Gene	CRHNA4		CRHNB2		
Mutation (aminoacid change)	c.839C > T (S280F)	c.870_872dupGCT (L291dup: insL)	c.851C > T (S284L)	c.859G > C (V287L)	
Cell type	*Xenopu*s oocyte	*Xenopu*s oocyte	*Xenopu*s oocyte	HEK293	*Xenopu*s oocyte
ACh sensitivity	Enhanced	Enhanced	Enhanced	Enhanced	Enhanced
Desensitisation	Enhanced	No	Enhanced	Reduced	Enhanced
Use‐dependent potentiation	Enhanced	Enhanced	No		
Ca^2+^ permeability	Reduced	Reduced	No	No	Reduced
Ca^2+^ dependency	Reduced	Reduced	Reduced	Reduced	Reduced
CBZ sensitivity (IC_50_: μM) (wild: 140 μM)	Enhanced (51 μM)	Enhanced (66 μM)	Reduced (296 μM)		
Reference IC_50_ of CBZ to wild‐type α4β2‐nAChR[1]	[2–9]	[2–4, 8–10]	[3, 8–11]	[12]	[8]

References: 1. Picard et al. (1999); 2. Figl et al. (1998); 3. Bertrand et al. (2002); 4. Bertrand et al. (1998); 5. Weiland et al. (1996); 6. Kuryatov et al. (1997); 7. Moulard et al. (2001); 8. Rodrigues‐Pinguet et al. (2003); 9. Rodrigues‐Pinguet et al. (2005); 10. Steinlein et al. (1997); 11. Matsushima et al. (2002); 12. De Fusco et al. (2000).

Subsequently, the functional abnormalities of ADSHE‐mutant nAChRs, including S284L and V287L mutations, were analysed using a wider range of parameters, such as desensitisation, Ca^2+^ permeability and Ca^2+^ dependency, as shown in Table 2 (Bertrand et al., 1998, 2002; Figl et al., 1998; Matsushima et al., 2002; Rodrigues‐Pinguet et al., 2003, 2005). Enhanced ACh sensitivity is a candidate common feature of ADSHE‐mutant α4β2‐nAChRs, but other parameters failed to detect commonalities (Bertrand et al., 2002; Figl et al., 1998; Rodrigues‐Pinguet et al., 2003, 2005). Extracellular Na^+^ and Ca^2+^ inflows through α4β2‐nAChR containing cation channels play important roles in the regulation of depolarisation, transmitter exocytosis and intracellular signalling (Figl et al., 1998; Fukuyama et al., 2020a; Rodrigues‐Pinguet et al., 2003). Reduced Ca^2+^ permeability of S280F‐, insL‐ and V287L‐mutant α4β2‐nAChRs was a direct impairment but was not observed in S284L‐mutant α4β2‐nAChR (Rodrigues‐Pinguet et al., 2003; Steinlein et al., 1997). However, S284L‐mutant α4β2‐nAChR reduced the total Ca^2+^ inflow due to decreased ACh sensitivity (Rodrigues‐Pinguet et al., 2003, 2005). Based on these findings, the abnormalities observed in the ADSHE‐mutant α4β2‐nAChR are probably due to loss of function, although the detailed mechanisms remain to be clarified.

In spite of these efforts, interpreting the previously published results regarding ADNFLE‐mutant α4β2‐nAChRs is difficult because of the very different experimental conditions (expression in HEK293 or *Xenopus* oocytes transfected by rodents or human clones). Moreover, some mutations, such as S280F (Bertrand et al., 2002) and V287L (De Fusco et al., 2000), show certain features under homozygous, but not heterozygous, conditions. The balance of subunits is probably also important, as α4β2‐nAChR can have (α4)_2_(β2)_3_ or (α4)_3_(β2)_2_ stoichiometries with different affinities to ACh. Indeed, the ACh sensitivity of (α4)_2_(β2)_3_‐nAChR is 100 times higher than that of (α4)_3_(β2)_2_‐nAChR (Moroni et al., 2006). Two candidate mechanisms of enhancement of ACh sensitivity of the ADNFLE‐mutant α4β2‐nAChR have been proposed and include an increase in the ACh sensitivity property of individual ADNFLE‐mutant nAChR subunits or a reduction of the proportion of low‐sensitivity (α4)_3_(β2)_2_‐nAChR in the population, because V287L‐mutation enhances the ACh sensitivities of both (α4)_2_(β2)_3_‐nAChR and (α4)_3_(β2)_3_‐nAChR but decreases the expression of low‐sensitivity (α4)_3_(β2)_2_‐nAChR (Nichols et al., 2016). Therefore, in vivo experiments using genetic rodent ADSHE models will probably provide other detailed pathomechanisms of ADNFLE.

From a different perspective, another achievement of single‐cell models was that some ADSHE mutations, such as S280F‐ and insL‐mutant α4β2‐nAChRs, displayed enhanced carbamazepine sensitivity (Bertrand et al., 2002; Picard et al., 1999), whereas the S284L‐mutant α4β2‐nAChR exhibited reduced carbamazepine sensitivity (Bertrand et al., 2002). The correlation between carbamazepine sensitivity with the clinical and electrophysiological features of ADSHE‐mutant α4β2‐nAChRs suggests that these mutations are possibly involved in the pathophysiology of ADSHE subclasses, even if it is unclear if the abnormality of ADSHE‐mutant α4β2‐nAChR results in a loss of function or gain of function (Bertrand et al., 2002).

## 4 FUNCTIONAL ABNORMALITIES IN RODENT MODELS

The transfected single‐cell model with the patch‐clamp method is a useful technique to elucidate the functional abnormalities of ion channels. However, neuroscientific experiments using epileptic animal models can identify the responsible neural circuits and clarify the various developmental processes of epileptogenesis/ictogenesis. Epileptic animal models are practical tools to explore the pathogenesis/pathophysiology of age‐dependent onset and can trigger event‐related features of genetic epilepsies. Nonetheless, the human brain is enormously complex, and even the most basic functions in rodents become difficult to integrate into a single plausible human model. Therefore, an interpretation of the experimental results obtained has no scientific value unless it is confirmed that the animal model is a reliable model of the human disorder.

### 4.1 Reliability and validation criteria of epilepsy animal models

Improving the reproducibility of preclinical experimental results is one of the most important elements when elucidating pathomechanisms and developing therapeutic agents. The validation criteria define whether the experimental animal model mimics the pathomechanisms of human disease, and the reliability criteria are defined for improving the inter‐investigator variations in data. Recently, the reliability criteria of epilepsy/convulsion models were reported by the joint translational task force of the International League Against Epilepsy and American Epilepsy Society (Barker‐Haliski et al., 2018; Harte‐Hargrove et al., 2018). Previous reports by multiple institutes confirmed the epileptic phenotypes and EEG of genetic ADSHE models. Indeed, the epileptic phenotype of S284L‐TG was detected using video‐EEG monitoring by three independent laboratories (Yamada et al., 2013; Zhu et al., 2008). The validation criteria of experimental animal models in neuropsychiatric fields have been proposed by a number of reports (Coenen & Van Luijtelaar, 2003; Fukuyama et al., 2020a; Nestler & Hyman, 2010; Okada et al., 2010; Stewart & Kalueff, 2015; Yamamura et al., 2013; Zhu et al., 2008). Unfortunately, there have been no consensus validation criteria for genetic epilepsy models, except for the spontaneous absence epilepsy models, GAERS and WAG/Rij (Coenen & Van Luijtelaar, 2003; Yamamura et al., 2013). This review only introduces our validation criteria, which are partial modifications of the validation criteria of Coenen and van Luijtelaar (Coenen & Van Luijtelaar, 2003; Fukuyama et al., 2020a; Okada et al., 2010; Yamamura et al., 2013; Zhu et al., 2008).

- **Face validity** of an ADSHE model is the ability to faithfully mimic the clinical symptoms of the disorder, including NPA, NPD, ENW and clustering during non‐REM sleep (Fukuyama et al., 2020a; Okada et al., 2010; Zhu et al., 2008).
- **Predictive validity** is the ability to predict previously unknown mechanisms of medication sensitivity of ADSHE, showing anticonvulsant sensitivity comparable to those of ADSHE patients (Fukuyama et al., 2020a; Okada et al., 2010; Zhu et al., 2008).
- **Construct validity** of ADSHE conforms to a theoretical rationale for genetic epilepsy, such as gene mutation, expression of mutant molecules or transmission abnormalities (Fukuyama et al., 2020a; Okada et al., 2010; Zhu et al., 2008).

Coenen and Van Luijtelaar (2003) listed the electroencephalographic features, typical spike‐and‐wave discharges, in the construct validity of absence epilepsy models, because the epileptic discharges in cortico‐thalamo‐cortical network play important roles in the generation of simultaneous onsets of phenotypic (absence seizure) and electroencephalographic (spike‐and‐wave discharge) phenomena in both patients and rodent models with absence epilepsy (Coenen & Van Luijtelaar, 2003). Contrary to absence epilepsy, the electroencephalographic features of ADSHE, behavioural abnormalities with epileptiform correlates (EEG‐sensitive) such as ENW and NPA, and without epileptiform correlates (EEG‐insensitive) such as dystonic‐arousal complex (DAC) and NPD and normal EEG background activities of ADSHE model, cannot contribute to comprehensive factor of construct validity of ADSHE model, because each electroencephalographic feature can merely interpret a part of pathophysiology of each respective ADSHE seizure subtypes. However, the detailed mechanisms of mutant α4β2‐nAChRs on these behavioural and electroencephalographic features of ADSHE remain to be clarified (K. Fukuyama, Fukuzawa, & Okada, 2020; K. Fukuyama, Fukuzawa, Okubo et al., 2020; K. Fukuyama et al., 2020a, 2020b; Fukuyama & Okada, 2020; Okada et al., 2010; Shiba et al., 2015; Yamamura et al., 2013; Zhu et al., 2008). Therefore, before analysis of the phenotype of the S284L‐TG and S286L‐TG models, we should consider the electroencephalographic features of ADSHE as aspects of face validity, rather than of construct validity, as in the absence epilepsy models.

The genetic mutations of ADSHE contribute to the fundamental roles in the pathomechanisms of ADSHE, due to the high penetrance in ADSHE pedigrees. Therefore, there is no doubt that genetic abnormalities are one of the major elements of construct validity in the ADSHE model. There are two types of genetic ADSHE models: transgenic rodents (mice and rats) and KI mice. Transgenic techniques are disadvantageous as transgene expression is not regulated by natural promoters. In contrast, KI mice have a more robust construct validity than transgenic rodents, although there is no evidence that the mouse brain can reflect the pathomechanisms of human epilepsy more closely than the rat brain (see Sections 4.2, 4.4). Furthermore, whether the mechanisms underlying other phenotypic features of ADSHE, such as age‐dependent onset and sleep‐related seizures, should be listed in construct validity or face validity, should be discussed in the future.

Predictive validity (or pharmacological validity) seems to be a highly vexed concept. Traditionally, one of the major targets of approved anticonvulsants, voltage‐dependent Na^+^ channels (VDSC), has been identified post hoc by studying the mechanism of action of drugs identified by serendipity. Additionally, absence seizures can be elicited by a relatively low dose of pentylenetetrazole in the absence epilepsy models, compared to healthy rodents, and this model has been used in the preclinical screening of putative antiepileptic drugs (Chen et al., 2011; Coenen & Van Luijtelaar, 2003). Therefore, pharmacological advances in the elucidation of pathophysiology can possibly identify the overlap of the components involved between predictive validity and construct validity. Although the three aspects of validity, face, construct and predictive validities, are established and cannot be changed or improved, the elucidation or discovery of the detailed pathogenesis or pathomechanisms can give new insights and this may cause the validity of the model being changed and/or being revised. Also when newly identified mechanisms of positive responses to therapeutic agents play an important role in the understanding of the pathogenesis or pathomechanisms of the disease, then the identified functional abnormalities (mechanisms of therapeutic response to therapeutic agents) should be reconsidered as no longer contributing to predictive validity but should be considered as contributing to construct validity. This is because the identified functional abnormality and its response to treatment might become a candidate target for development of novel medications.

### 4.2 S280F‐ and insL‐mutant models

Two KI animal models harbouring the same mutant *Chrna4* gene, corresponding to the human S280F‐mutant *CRHNA4*, have been generated and are known as S252F‐KI (Klaassen et al., 2006) and S248F‐KI (Teper et al., 2007) (Table 3). S252F‐KI and S248F‐KI acquired the same amino acid change in the α4‐nAChR subunit, but the ES cell lines used for the generation of S252F‐KI and S248F‐KI were different; J1 and W9.5 ES cells, respectively (Klaassen et al., 2006; Teper et al., 2007). One KI mouse model harbouring the mutant *Chrna4*, corresponding to the human insL‐mutant *CRHNA4*, was also generated using J1 ES cells, named insL‐KI, (Klaassen et al., 2006) (Table 3).

**TABLE 3:** Functional abnormalities of rodent models of ADSHE

Gene				CRHNA4			CRHNB2		
Mutation		c.839C > T (S280F)		c.870_872dupGCT (L291dup: insL)	c.851C > T (S284L)		c.859G > C(V287L)		
Animal model	(amino‐acid change)	S252F‐KI	S248F‐KI	insL‐KI	S284L‐TG	S286L‐TG	V287L‐TG	V287L‐KI	V286L‐TG
Construct validity		CRHNA4‐S280F knock‐in	CRHNA4‐S280F knock‐in	CRHNA‐865‐873insGCT knock‐in	CRHNA4‐S284L transgenic	CRHNA4‐S284L transgenic	CRHNB2‐V287L transgenic	CRHNB2‐V287L knock‐in	CRHNB2‐V287L transgenic
		(J1 ES cell line)	(W9.5 ES cell line)	(J1 ES cell line)	(PDGF‐β promotor)	(Chrna4 promotor)	(TET‐OFF system)	(R1 ES cell line)	(PDGF‐β promotor)
	Genetic background	C57BL/6 J	C57BL/6	C57BL/6 J	Sprague–Dawley rat	Sprague–Dawley rat	FVB	C57BL/6	Sprague–Dawley rat
		129S4/SvJae	129S4/SvJae	129S4/SvJae					
Face validity	Background EEG	Increase slow wave	Normal	Increase slow wave	Normal	Normal	Increase slow wave		Normal
	Spontaneous epileptic seizure	Motor seizure (wakefulness)	Non	Motor seizure (wakefulness)	ADSHE (NPA, NPD, ENW) during non‐REM	ADSHE (NPA, NPD, ENW) during non‐REM	Frequent ictal discharge (non‐REM > wakefulness)	Non	ADSHE (NPA) during non‐REM
	Behaviour				Reduction social interaction time			Disturbances in the normal sleep patterns	
					Increased activity level in novel environment			Anxiety‐related behaviour (increased activity level in novel environment)	
Predictive validity	Induced seizures	Shorter latencies to onset and longer seizure durations of nicotine‐induced seizure than wild type	Nicotine‐induced EEG‐insensitive DAC		Nicotine (4 mg kg^‐1^) induced partial seizure but not generalised seizure			Disturbed sleep pattern and circadian rhythm	Nicotine (0.5 and 1 mg kg^‐1^) induced seizure
		Sub‐proconvulsive dose of picrotoxin (0.1 mg kg^‐1^) inhibis spontaneous seizures	Normal EEG background during DAC		Less severe nicotine‐induced seizures than wild type			Nicotine‐induced EEG‐insensitive DAC	Shorter latencies to onset and longer seizure durations of nicotine‐induced seizure than wild type
			Less severe nicotine‐induced seizures than wild type		Almost equal PTZ sensitivity			After DAC, EEG‐sensitive tonic–clonic seizure	
	Anticonvulsant sensitivity		CBZ inhibits DAC		CBZ no effect on interictal discharge ZNS and DZP decrease interictal discharge (45%)	CBZ no effect on interictal discharge ZNS decrease interictal discharge (70%)	CBZ no effect on seizure frequency and duration, whereas CBZ supresses burst of cultured cortical neurones of V287L‐TG		CBZ no effect on ictal frequency and duration
Others		Enhanced GABAergic inhibition induced by nicotine			Impaired reduction of glutamate release during sleep		Silencing V287L‐Chrna4 during embryonic state suppresses seizure severity	Reduced levels of anxiety	
					Impaired synaptic and extrasynaptic GABAergic inhibition induced by nicotine		Silencing V287L‐Chrna4 after seizure onset unaffect seizure severity	Abnormal natural reward	
					Chronic furosemide administration prevents ADSHE onset				
Reference		[1]	[2]	[1]	[3, 4]	[5–9]	[10, 11]	[12, 13]	[14]

References: 1. Klaassen et al. (2006); 2. Teper et al. (2007); 3. Zhu et al. (2008); 4. Yamada et al. (2013); 5. Fukuyama, Fukuzawa, and Okada (2020); 6. K. Fukuyama, Fukuzawa, Okubo et al., (2020); 7. Fukuyama et al., (2020a); 8. Fukuyama et al., (2020b); 9. Fukuyama and Okada (2020); 10. Manfredi et al. (2009); 11. Gullo et al. (2014); 12. Xu et al. (2011); 13. O'neill et al. (2013); 14. Shiba et al. (2015).

The phenotypes of S252F‐KI and insL‐KI are quite similar (Klaassen et al., 2006). Both KI mice displayed markedly increased slow waves (0.5–4 Hz) in background EEG and spontaneous epileptic seizures during wakefulness (Klaassen et al., 2006). Both S252F‐KI and insL‐KI showed important pharmacological features. These two KI mice displayed enhanced sensitivity to nicotine application, because their nicotine‐induced seizures showed shorter latencies to seizure onset and longer durations than the wild type (Klaassen et al., 2006). The slice patch‐clamp method demonstrated that nicotine application did not affect the excitatory synaptic current (EPSC) but markedly activated the inhibitory postsynaptic current (IPSC) in layers II/III of the cortex. In contrast, spontaneous EPSC and IPSC were not affected. These results suggest that the enhanced GABAergic inhibition induced by S280F‐mutant and insL‐mutant α4β2‐nAChRs play important roles in ADSHE epileptogenesis/ictogenesis with S280F and insL mutations (Klaassen et al., 2006).

In contrast, another S280F KI mouse model, S248F‐KI (harbouring the same mutation of S252F‐KI corresponding to the human S280F mutation but produced using a different W9.5 ES cell line) displayed lower sensitivity to nicotine‐induced seizures, without spontaneous seizures or EEG background abnormality. However, nicotine application generated an EEG‐insensitive behavioural abnormality, the dystonic‐arousal complex (DAC), which consists of components of NPD with NPA (Teper et al., 2007) (Table 3). The nicotine‐induced EEG‐insensitivity of DAC suggests that activation of S280F‐mutant α4β2‐nAChR contributes to the pathomechanism of the EEG‐insensitive NPD‐like phenotype, DAC. Therefore, the face validities of S248F‐KI, S252F‐KI and insL‐KI were partly verified, but these imply limited face validities, because the outcomes of the verification study did not show an agreement between the model and what is clinically seen.

### 4.3 V287L mutant models

Three genetic animal models harbouring mutant *Chrnb2*, corresponding to the human V287L‐mutant *CRHNB2*, have been generated (Gullo et al., 2014; Manfredi et al., 2009; Shiba et al., 2015; Xu et al., 2011) (Table 3). One type of transgenic mouse, named V287L‐TG, was developed employing a tetracycline‐controlled promoter, which allowed the researchers to silence the mutated gene in a reversible fashion (TET‐OFF system) (Manfredi et al., 2009). The frequency of spontaneous interictal/ictal discharges was increased in the V287L‐mutant in a gene expression‐dependent manner (10‐fold increased β2‐nAChR compared to wild type) (Manfredi et al., 2009), but the spontaneous seizure frequency during non‐REM sleep was higher than during wakefulness (Manfredi et al., 2009). Thus, the face validity of V287L‐TG was only partly verified. Furthermore, V287L‐mutant α4β2‐nAChR may affect the severity of spontaneous seizure expression in an expression level‐dependent manner. However, after the onset of a spontaneous seizure, silencing of the V287L‐mutant gene could not prevent further seizures. In contrast, blockade of V287L‐mutant gene expression during embryonic day 1 to postnatal day 15 prevented spontaneous seizures and EEG abnormalities. Thus, overexpression of the V287L‐mutant β2‐nAChR probably contributes to the epileptogenesis of ADSHE, although this is not related to ictogenesis (Table 3) (Manfredi et al., 2009). The other KI mouse model, named V287L‐KI, created using the R1 ES cell lines, displayed an altered sleep pattern and nicotine‐induced DAC (similar to S248F‐KI), without spontaneous seizures (O'neill et al., 2013; Xu et al., 2011). The behaviour of V287L‐KI mice was generally normal (O'neill et al., 2013; Xu et al., 2011), but abnormalities such as anxiety and natural reward (nicotine behavioural addiction: determination using wheel‐running activity), associated with the α4β2‐nAChR of V287L‐KI, were detected (Table 3) (Xu et al., 2011). Transgenic rats carrying V286L‐TG mutant *Chrnb2*, corresponding to the human V287L‐mutant *CRHNB2*, display EEG‐sensitive NPA (45%) without other ADSHE seizures (NPD and ENW), although they also exhibited hypersensitivity to nicotine‐induced seizures (Table 3) (Shiba et al., 2015). Therefore, the face validities of V287L‐KI, V287L‐TG and V286L‐TG were also partly verified, but these imply limited face validities, because the outcomes of the verification study did not show an agreement between the model and clinical observation. However, V287L‐TG indicated the important pathomechanism of ADSHE with V287L‐mutation, because the V287L‐mutant β2‐nAChR subunit possibly contributes to the generation of abnormal/epileptic formation of neuronal circuits and/or long‐lasting alterations in network assembly during the developing brain.

Carbamazepine was effective at controlling ADSHE seizures in all patients with V287L‐mutation (Gambardella et al., 2000), whereas the seizure frequency of V287L‐TG could not be reduced by carbamazepine (Manfredi et al., 2009). Contrary to the poor response to carbamazepine in V287L‐TG, neuronal firing of primary cultured neocortical neurons of V287L‐TG using a multi‐electrode array revealed that the inhibitory effects of carbamazepine on neuronal firing of V287L‐TG was more dominant than that of the wild type (Gullo et al., 2014). Therefore, the predicted pharmacological validity of V287L‐TG was also partly verified, but this implies limited predictive validity, because carbamazepine, which is a first‐line anticonvulsant for the treatment of patients with ADSHE with V287L‐mutation (Gambardella et al., 2000), suppresses neuronal activity (Gullo et al., 2014) but cannot decrease seizure frequency in V287L‐TG (Manfredi et al., 2009).

### 4.4 S284L mutant models

Two transgenic rat models harbouring mutant *Chrna4*, corresponding to human S284L‐mutant *CRHNA4*, named S284L‐TG and S286L‐TG, were generated (Table 3) (K. Fukuyama, Fukuzawa, & Okada, 2020; K. Fukuyama, Fukuzawa, Okubo et al., 2020; Fukuyama et al., 2020a, 2020b; Fukuyama & Okada, 2020; Yamada et al., 2013; Zhu et al., 2008). The promoters used in S284L‐TG and S286L‐TG were rat PDGF‐β and wild‐type *Chrna4*, respectively, but their face and predictive validities were verified (Fukuyama et al., 2020b; Zhu et al., 2008). In particular, both rat models displayed the three distinct ADSHE seizures, ENW, NPA and NPD, spontaneously during non‐REM sleep, without any EEG background abnormalities, and expressed carbamazepine‐insensitive with zonisamide‐sensitive anticonvulsant susceptibility, resembling ADSHE patients with the S284L‐mutation (Fukuyama et al., 2020b; Zhu et al., 2008). Although the S284L‐TG and S286L‐TG phenotypes fulfil the face validity, the frequency of ADSHE seizures in these models is lower than that in untreated ADNFLE patients (once a week seizure frequency) (Fukuyama et al., 2020b; Zhu et al., 2008). In S286L‐TG, clustering is rare, but seizures are observed multiple times a day (Fukuyama et al., 2020a). Additionally, the onset of interictal and ictal discharges occurred at 6 and 8 weeks of age, respectively (Fukuyama & Okada, 2020; Yamada et al., 2013; Zhu et al., 2008).

#### 4.4.1 Transmission abnormalities in S284L‐TG and S286L‐TG

Various transmission abnormalities have been demonstrated in S284L‐TG (Yamada et al., 2013; Zhu et al., 2008). Before ADSHE onset, abnormalities in the basal release of glutamate and GABA, expression of the sodium–potassium chloride co‐transporter NKCC1 and the potassium chloride co‐transporters KCC1 or KCC2, were not observed, whereas impaired α4β2‐nAChR‐induced synaptic and extrasynaptic GABAergic transmission was detected. In contrast, after ADSHE onset, basal frontal glutamate release was relatively enhanced compared to that of the wild type, in the transition from wakefulness to non‐REM sleep (Zhu et al., 2008). S284L‐TG also represented the epileptogenic functional shifts, higher basal glutamate release (Zhu et al., 2008), increased NKCC1 and decreased KCC1/KCC2 expression, compared to the wild type (Yamada et al., 2013). Based on these functional abnormalities during the critical period for ADSHE onset in S284L‐TG, S284L‐mutant α4β2‐nAChR or its induced GABAergic disinhibition lead to epileptogenesis. Indeed, chronic furosemide (NKCC1 inhibitor) administration from 4 to 8 weeks of age prevented ADSHE onset in 67% of S284L‐TG rats (Yamada et al., 2013). In contrast, the remission rate with chronic furosemide administration after ADSHE onset (between 8 and 10 weeks of age) was only 37% (Yamada et al., 2013). In spite of these efforts, whether the increased frontal extracellular glutamate level in S284L‐TG was a functional abnormality associated with epileptogenesis/ictogenesis or a result of the acquisition of epileptogenesis/ictogenesis could not be clarified.

**FIGURE 1:**
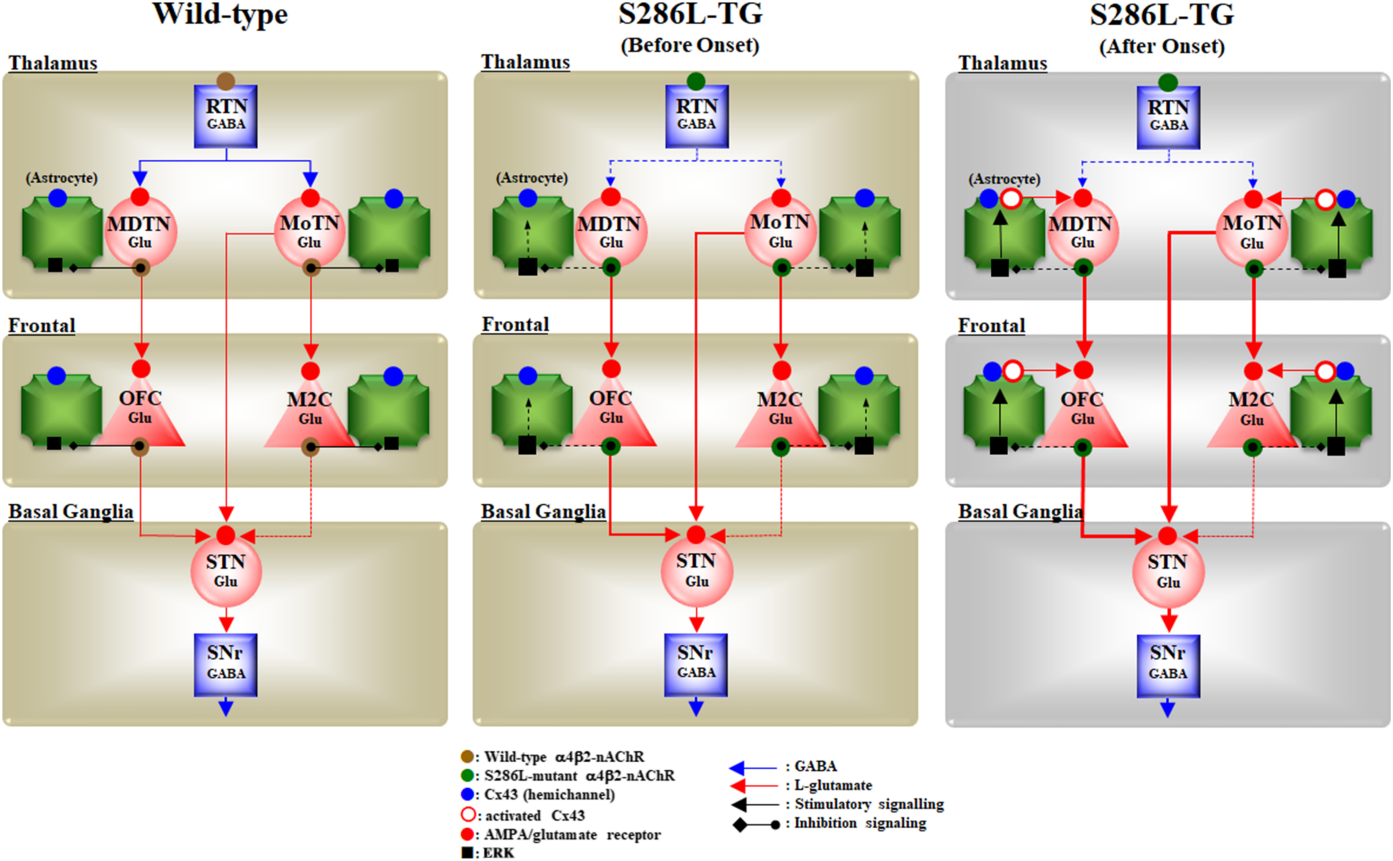
Scheme of proposed hypothesis of pathophysiology/ictogenesis of ADSHE of S286L‐TG. Proposed hypothesis of the pathophysiology of ADSHE of S286L‐TG associated with functional abnormalities of glutamatergic transmission in the thalamocortical and hyperdirect pathways in the wild type (a) and prior (b) and after (c) ADSHE‐onsets of S286L‐TG. The reticular thalamic nucleus (RTN) mainly projects GABAergic terminals to various thalamic nuclei, including the mediodorsal thalamic nucleus (MDTN) and motor thalamic nuclei (MoTN). The activation of α4β2‐nAChR in the RTN enhances GABAergic transmission in the RTN‐MDTN and RTN‐MoTN pathways of the wild type (panel a), whereas the S286L‐mutant α4β2‐nAChR impairs the activation of GABAergic transmission in the RTN‐MDTN in S286L‐TG (panels b and c). MDTN project glutamatergic terminals to the OFC. In the MDTN, both α4β2‐nAChR and the AMPA/glutamate receptor activate glutamatergic transmission to the OFC (panels a–c). Wild‐type α4β2‐nAChR inhibits astroglial ERK, resulting in the suppression of connexin43 expression in the astroglial plasma membrane (panel a). Contrary to the wild type, in S286L‐TG, the loss‐of‐function S286L‐mutant α4β2‐nAChR lacks suppressive effects on p‐ERK (panels b and c) but is insufficient to upregulate connexin43 (panel b). A combination of the persistent/repetitive propagation of the hyperactivation of glutamatergic transmission in MDTN‐OFC induced by the GABAergic disinhibition of S286L‐TG and p‐ERK upregulation enhances connexin43 expression

Various transmission abnormalities in S286L‐TG have also been identified (K. Fukuyama, Fukuzawa, & Okada, 2020; K. Fukuyama, Fukuzawa, Okubo et al., 2020; Fukuyama et al., 2020a, 2020b; Fukuyama & Okada, 2020). During the interictal stage, basal extracellular glutamate levels in the secondary motor cortex (M2C), orbitofrontal cortex (OFC), mediodorsal thalamic nucleus (MDTN) and motor thalamic nuclei (MoTN) of S286L‐TG were higher than those of wild type (K. Fukuyama, Fukuzawa, & Okada, 2020; K. Fukuyama, Fukuzawa, Okubo et al., 2020; Fukuyama et al., 2020a, 2020b; Fukuyama & Okada, 2020). These regions in the thalamus and frontal cortex are the predominant expression regions of α4‐nAChR (Fukuyama et al., 2020b; Zhu et al., 2008). Furthermore, the stimulatory effects of α4β2‐nAChR on GABAergic neurones in the reticular thalamic nucleus (RTN) were impaired in S286L‐TG, resulting in partial intrathalamic GABAergic disinhibition (Figure 1) (Fukuyama et al., 2020b). This alteration enhanced thalamocortical glutamatergic transmission (Fukuyama et al., 2020b). Interestingly, neither the M2C nor OFC can generate epileptic discharges independently, althoughthey can integrate external excitatory inputs from the thalamocortical pathway, resulting in the generation of epileptic discharges in these regions (Figure 1) (K. Fukuyama, Fukuzawa, & Okada, 2020; K. Fukuyama, Fukuzawa, Okubo et al., 2020; Fukuyama et al., 2020b; Fukuyama & Okada, 2020).

Enhanced glutamatergic transmission in the thalamo‐subthalamic pathway, from the MoTN to the subthalamic nucleus (STN), was also observed in S286L‐TG (Figure 1) (Fukuyama et al., 2020b). Therefore, hyperactivation of glutamatergic transmission in the thalamo‐subthalamic pathway contributes to the generation of EEG‐insensitive tonic/dystonic or hyperkinetic behaviour (Klaassen et al., 2006; O'neill et al., 2013; Xu et al., 2011). This hyperactivation of the thalamo‐subthalamic pathway suggests that hyperactivation of the MoTN, induced by ADSHE‐mutant nAChRs, probably propagates predominantly to the basal ganglia rather than the frontal cortex at least, during NPD (Figure 1) (Fukuyama et al., 2020b). In spite of these efforts, increased basal extracellular glutamate levels in the MoTN, MDTN, STN, M2C and OFC of S286L‐TG could not be fully explained by intrathalamic GABAergic disinhibition, as intrathalamic GABAergic transmission is phasic inhibition (Okada, Fukuyama, Kawano, et al., 2019).

#### 4.4.2 Upregulated/activated mCx43 in S286L‐TG

The mechanisms of generation of ADSHE foci, clustering and complications among ENW, NPA and NPD in the same episode, are not adequately explained by intrathalamic GABAergic disinhibition alone. However, it can be reasonably interpreted by other functional abnormalities of the tripartite synaptic transmission (Figure 1) (K. Fukuyama, Fukuzawa, & Okada, 2020; K. Fukuyama, Fukuzawa, Okubo et al., 2020; Fukuyama & Okada, 2020). After ADSHE onset, connexin43 (Cx43) in the plasma membrane (mCx43) was upregulated in both the thalamus and frontal cortex, whereas before onset of interictal and ictal discharges (4 weeks of age), mCx43 expression in the frontal cortex was not upregulated (K. Fukuyama, Fukuzawa, & Okada, 2020; K. Fukuyama, Fukuzawa, Okubo et al., 2020; Fukuyama & Okada, 2020).

Cx43 is the most widely and predominantly expressed connexin isoform in the brain, including astrocytes (Okada et al., 2021; Okada, Fukuyama, et al., 2020). Six connexin units assemble to form homomeric or heteromeric connexons. Two connexons in two neighbouring cells form a gap junction, which contributes to the cytoplasm‐to‐cytoplasm communication of biochemical and ionic mobilisation between adjacent cells, leading to the regulation of ionic and several other types of homeostasis, including regulation of intracellular Ca^2+^ mobilisation and K^+^ buffering (Okada et al., 2021; Okada, Fukuyama, et al., 2020). Single connexons contribute to the chemical connection between intra‐ and extracellular spaces as a hemichannel (Okada et al., 2021; Okada, Fukuyama, et al., 2020). During the resting stage, astrocytes are characterised by a high level of gap‐junctional communication but low hemichannel permeability (Okada et al., 2021; Okada, Fukuyama, et al., 2020). In contrast, pathological hyperactivated conditions, such as depolarisation, ischaemia, specific cation mobilisation and phosphorylation, generate persistent hemichannel opening, resulting in the persistent astroglial non‐exocytotic release of excitatory glutamate, D‐serine, ATP, kynurenine metabolites and eicosanoids, which leads to the disruption of several homeostasis systems (Okada et al., 2021; Okada, Fukuyama, et al., 2020).

**FIGURE 2:**
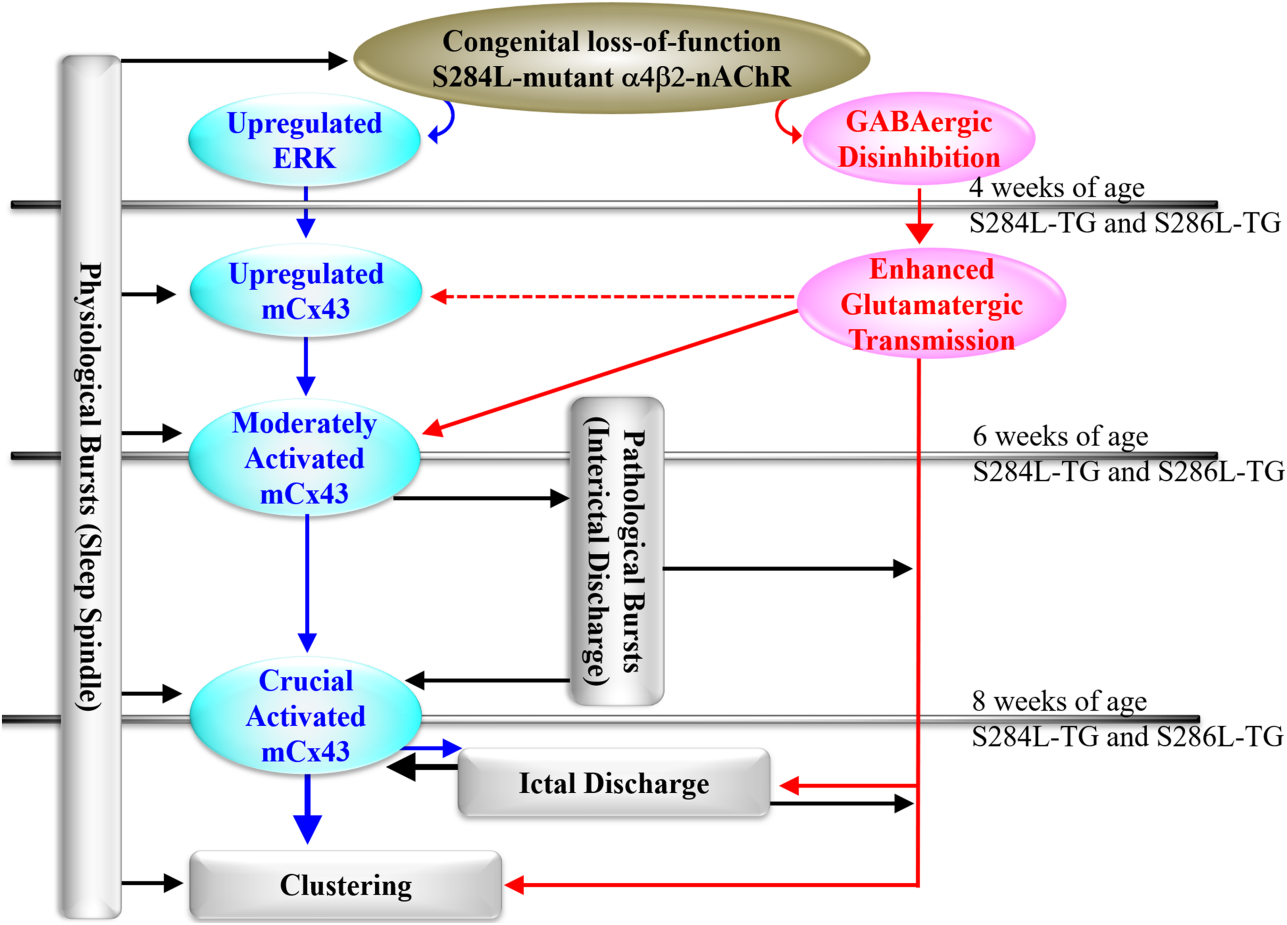
Scheme of proposed hypothesis of age‐dependent and event‐related pathomechanism/epileptogenesis of ADSHE of S286L‐TG. Proposed hypothesis of age‐dependent and event (sleep and seizure)‐related pathomechanism/epileptogenesis associated with connexin43 and S286L‐mutant α4β2‐nAChR of S286L‐TG. Before ADSHE onset (until 4 weeks of age), loss‐of‐function S286L‐mutant α4β2‐nAChRs generate upregulation of ERK signalling and intrathalamic GABAergic disinhibition, resulting in weakly enhanced glutamatergic transmission in the thalamocortical and thalamic hyperdirect pathways. During 4 and 8 weeks of age (critical period for interictal discharge onset), upregulated ERK signalling gradually increases expression of connexin43 in the astroglial plasma membrane. The upregulated astroglial connexin43 hemichannels are activated by both physiological sleep spindle and pathological interictal discharges. At the critical ADSHE onset period (8 weeks of age), the combination of accumulating physiological and pathological bursts and upregulated connexin43 hemichannels leads to enhanced tripartite synaptic transmission, resulting in the generation of ADSHE ictal discharges in the regions where expression of α4β2‐nAChRs is predominant

Upregulated mCx43 in the thalamus and frontal cortex of S286L‐TG has already been shown to be weakly but functionally activated during the interictal stage (K. Fukuyama, Fukuzawa, & Okada, 2020; K. Fukuyama, Fukuzawa, Okubo et al., 2020; Fukuyama & Okada, 2020), resulting in increased basal L‐glutamate release in the frontal cortex of both S284L‐TG and S286L‐TG (Figures 1 and 2) (Fukuyama et al., 2020b; Zhu et al., 2008). The lack of change in glutamate release during the transition from wakefulness to non‐REM sleep of S284L‐TG (Zhu et al., 2008) can also be considered a possible event, as glutamate release occurs via predominantly non‐exocytotic mCx43 release rather than neuronal exocytosis. The regions of upregulated mCx43 corresponded to the α4β2‐nAChR predominant expression regions (thalamus and frontal cortex) of S286L‐TG. This association provides more detailed information regarding the pathomechanisms of ADSHE from the S284L‐mutation, via ligation of fragmentary findings associated with epileptogenesis (K. Fukuyama, Fukuzawa, & Okada, 2020; K. Fukuyama, Fukuzawa, Okubo et al., 2020; Fukuyama & Okada, 2020; Fukuyama, Ueda et al., 2020).

The electrophysiological inputs in the thalamus, including RTN, MoTN and MDTN, activate astroglial hemichannel activities, leading to enhancement of thalamo‐subthalamic and thalamocortical glutamatergic transmission (Okada, 2019; Okada, Fukuyama, Kawano, et al., 2019; Okada, Fukuyama, Nakano, & Ueda, 2019; Okada, Fukuyama, Okubo, et al., 2019; K. Fukuyama, Fukuzawa, & Okada, 2020; K. Fukuyama, Fukuzawa, Okubo et al., 2020; Fukuyama et al., 2020a; Fukuyama et al., 2020b; Fukuyama & Okada, 2020). Enhanced thalamo‐subthalamic glutamatergic transmission produces an imbalance in the transmission of the basal ganglia (Fukuyama et al., 2020b). The integration of enhanced thalamocortical inputs (primarily through the MoTN‐M2C pathway), on electrophysiology, seem to generate an epileptic discharge (Fukuyama et al., 2020b). In fact, the accumulation of repetitive excitatory thalamocortical inputs reaches the threshold of astroglial hemichannel activation, resulting in the generation of epileptic bursts in M2C (Figures 1 and 2). The OFC is considered one of the dominant focus regions (Provini et al., 1999), although the OFC stimulation induced by intrathalamic GABAergic disinhibition was weaker than that of M2C (Fukuyama et al., 2020a; Fukuyama & Okada, 2020). Contrary to the interictal stage, the propagation of sustained/repetitive hyperexcitability in the MDTN or OFC activated regional hemichannels (K. Fukuyama, Fukuzawa, & Okada, 2020; Fukuyama & Okada, 2020). Persistent glutamate release through activated astroglial hemichannels in MDTN and OFC enhanced glutamatergic transmission in MDTN‐OFC, leading to the generation of ADSHE foci in the OFC (Figures 1 and 2) (K. Fukuyama, Fukuzawa, & Okada, 2020; K. Fukuyama, Fukuzawa, Okubo et al., 2020; Fukuyama & Okada, 2020). Therefore, persistent/repetitive excitabilities, including the propagation of sleep spindle or pathological (interictal/ictal discharges) bursts, activate the function of upregulated mCx43 in the thalamus and frontal cortex. This activation of mCx43 leads to the generation of ADSHE foci in the frontal cortex and, at least partly, complications between EEG‐insensitive and EEG‐sensitive behaviours of ADSHE. Furthermore, the interesting clinical features, such as clustering or frequent ADSHE seizures during the same night after experiencing the first seizure (even if the ADSHE seizures are controlled for long periods of time), are also likely to be caused by the activation of upregulated mCx43. Therefore, the hyperexcitable tripartite synaptic transmission, associated with upregulated/activated mCx43, promotes the ictogenesis of ADSHE clustering, because hemichannel activation can last for several hours (Figures 1 and 2) (K. Fukuyama, Fukuzawa, & Okada, 2020; K. Fukuyama, Fukuzawa, Okubo et al., 2020; Fukuyama, Ueda et al., 2020).

#### 4.4.3 mCx43 and S286L‐TG

In the focus regions of partial epilepsy patients and experimental animal models, including S286L‐TG, mCx43 is upregulated in astrocytes, whereas similar activation is not observed in the neurons (Das et al., 2012; K. Fukuyama, Fukuzawa, & Okada, 2020; K. Fukuyama, Fukuzawa, Okubo et al., 2020; Garbelli et al., 2011; Hussein et al., 2016; Walrave et al., 2020). It is well known that astroglial hemichannels play important roles in epileptogenesis/ictogenesis under subclinical proinflammatory responses (Fukuyama & Okada, 2018; Medina‐Ceja et al., 2019). Indeed, hemichannel inhibitors can prevent the onset of epileptic seizures (Li, Li, et al., 2019). The combination of intrathalamic GABAergic disinhibition with enhanced tripartite transmission probably contributes to the pathomechanisms of ADSHE with S284L mutation. Therefore, identifying the implication of whether the upregulated/activated mCx43 is the pathomechanisms of epileptogenesis/ictogenesis of ADSHE or a phenomenon as a result of acquisition of epileptogenesis/ictogenesis in S286L‐TG is a fundamental scientific issue, because before the onset of interictal/ictal discharges (4 weeks of age), the expression of mCx43 in S286L‐TG is almost equal to that of the wild type (Fukuyama & Okada, 2020).

ADSHE onset in S284L‐TG can be prevented by chronic administration of the NKCC1‐inhibitor furosemide (Yamada et al., 2013). This experiment was based on the findings that upregulation of NKCC1 after ADSHE onset in S284L‐TG, which was not observed before ADSHE onset (Yamada et al., 2013). However, the pharmacodynamic profile of furosemide suggests the presence of other pathomechanisms, because furosemide inhibits mitogen‐activated protein kinase/extracellular signal‐regulated kinase (MAPK/ERK) signalling (Panet et al., 2006). MAPK/ERK signalling regulates protein phosphorylation and cell functions, such as proliferation, division, differentiation, survival and apoptosis (Fukuyama & Okada, 2020; Okada et al., 2021; Okada, Fukuyama, et al., 2020; Okada, Kawano, et al., 2020). Transport to the plasma membrane, intracellular communication and degradation of Cx43 are regulated by post‐transcriptional processes (Fukuyama & Okada, 2020; Okada, Fukuyama, et al., 2020; Ribeiro‐Rodrigues et al., 2017). Activated MAPK/ERK and PI3K/Akt signalling increases the expression of mCx43 without affecting mRNA (Cushing et al., 2005; Fukuyama & Okada, 2020; Lin et al., 2013; Okada, Fukuyama, et al., 2020). Indeed, before ADSHE onset, the MAPK/ERK signalling of S286L‐TG was upregulated, whereas Akt signalling was almost equal to that of wild type (Fukuyama & Okada, 2020). Furthermore, sub‐chronic nicotine administration suppressed mCx43 and ERK signalling but enhanced Akt signalling in wild‐type cells, whereas sub‐chronic nicotine administration enhanced Akt signalling but did not affect mCx43 expression or Erk signalling in S286L‐TG (Fukuyama & Okada, 2020). These results indicate that loss‐of‐function of the ADNFLE‐mutant α4β2‐nAChR also possibly has loss‐of‐function of suppressive effects on ERK signalling, leading to upregulation of mCx43.

The increased basal glutamate release of S286L‐TG compared to the wild type is also important as the pathomechanism of ADSHE, according to the imbalance hypothesis of epilepsy (Hirose et al., 2000). During the resting stage, astroglial gap junctions act as permeable intracellular molecules via functionally opening probability, whereas astroglial hemichannel exhibits low opening probability (Jeanson et al., 2015; Liu et al., 2016). Based on these experimental findings, we believe that astroglial hemichannels do not contribute to gliotransmitter release during the resting state due to their low opening probability (K. Fukuyama, Fukuzawa, & Okada, 2020; K. Fukuyama, Fukuzawa, Okubo et al., 2020; Fukuyama & Okada, 2020; Fukuyama, Ueda et al., 2020; Jeanson et al., 2015; Liu et al., 2016). However, currently, we speculate that even in the low opening probability, the upregulation of mCx43 is probably involved in the increased basal release of glutamate in S286L‐TG during the interictal state, because astroglial hemichannel inhibitors unexpectedly decreased the increased basal L‐glutamate release of S286L‐TG, without affecting that of wild type (K. Fukuyama, Fukuzawa, & Okada, 2020; K. Fukuyama, Fukuzawa, Okubo et al., 2020). Therefore, it is undeniable that the increased basal release of glutamate in S286L‐TG is provided by glutamate released through astroglial hemichannels.

Previous studies have found that neurotransmitter release requires extracellular K^+^ concentrations of greater than 25 mM, whereas astrocytes require a concentration greater than 100 mM (K. Fukuyama, Fukuzawa, & Okada, 2020; Kawata et al., 1999). Physiological neural activity increases extracellular K^+^ levels up to 1 mM, whereas epileptic discharges increase extracellular K^+^ levels to around 10–12 mM (Carmignoto & Haydon, 2012). Furthermore, mCx43 expression in primary cultured astrocytes is increased by exposure to sustained K^+^ levels higher than 10 mM for several hours (Fukuyama & Okada, 2020). Considering that activated hemichannels release K^+^ into the extracellular space, activated hemichannels induced by elevation of extracellular levels of excitatory transmitter and depolarisation via sleep spindles and interictal/ictal discharges increase further elevation of extracellular K^+^ levels and various excitatory gliotransmitters, leading to the possible generation of a persistent self‐enhancing cycling of hemichannels. Therefore, it should be clarified to what extent the exposure to 10 mM K^+^ for several hours, as shown by in vitro experiments, reflects the activation of astroglial hemichannel process in the focus region of S286L‐TG. Specifically, confirming that the response to astroglial hemichannel activation by exposure for several minutes to several tens of minutes, provides a more detailed understanding of the involvement of upregulated mCx43 in ictogenesis in S286L‐TG.

These channels are also involved in memory formation/consolidation, whereas several preclinical behavioural studies have reported that both impaired and hyperactivated astroglial functions contribute to cognitive disturbance, such as loss of short‐term spatial memory and fear memory consolidation (He et al., 2020). Interestingly, a post mortem study demonstrated an upregulation of Cx43 in the frontal cortex of patients with autism compared to healthy subjects (Fatemi et al., 2008). Therefore, upregulated mCx43 around epileptic foci or neural circuits for the propagation of discharges is critical for the clinical features of epileptic seizures and comorbid cognitive impairment of ADSHE patients with S284L mutation.

#### 4.4.4 Pathophysiology of carbamazepine‐resistant/zonisamide‐sensitive ADSHE with the S284L mutation

The major anticonvulsive mechanisms of carbamazepine and zonisamide are considered to be quite similar and involve the inhibition of VDSC (Sills & Rogawski, 2020). However, the antiepileptic spectrum of each anticonvulsant should be considered as the integration between the inhibition of spreading epileptic hyperexcitability via VDSC inhibition and the effects on other transmission‐regulating systems (Fukuyama, Ueda et al., 2020; Okada et al., 2002; Sills & Rogawski, 2020; Yamamura et al., 2009). Therapeutically relevant concentrations of zonisamide acutely inhibit astroglial hemichannel activity and chronically suppress mCx43 expression, but carbamazepine has no similar effects (K. Fukuyama, Fukuzawa, & Okada, 2020; K. Fukuyama, Fukuzawa, Okubo et al., 2020; Fukuyama, Ueda et al., 2020). Furthermore, carbamazepine, which is an adenosine A_2A_ receptor agonist (Okada et al., 1997; Okada, Fukuyama, Shiroyama et al., 2019), acutely enhances astroglial glutamate transmission due to its A_2A_ receptor agonistic action (Okada, Fukuyama, Shiroyama et al., 2019). Considering the pathophysiology of carbamazepine‐resistant/zonisamide‐sensitive ADSHE seizures with the S284L mutation, the discrepant effects of these anticonvulsants on the mCx43 hemichannel are a reasonable finding. However, the mechanisms of the suppressive effect of zonisamide on mCx43 expression are not modulated by MAPK/ERK signalling (Takaku & Sango, 2020). It has been reported that zonisamide affects various transmitter regulation systems, including the candidate targets for the suppression of mCx43 expression, such as ubiquitin ligase, metabotropic glutamate receptors and carbonic anhydrase [Yamamura et al., 2009; Omura et al., 2013; Fukuyama et al., 2014]). Further research is needed to clarify the specific mechanisms underlying the inhibitory effect of zonisamide on astroglial hemichannel expression.

## 5 REMAINING CHALLENGES

### 5.1 Effects of nAChRs on the intracellular signalling pathway

The mechanism through which the loss‐of‐function S286L‐mutant α4β2‐nAChR increases mCx43 is one of the fundamental scientific issues associated with ADSHE pathomechanism (Figure 2). The primary functions of the nAChR family are ligand‐gated cation channels. The nAChRs containing the α7 subunit (α7‐nAChRs) is less sensitive to ACh (EC_50_ is micromolar order) and exhibits rapid desensitisation (in the order of milliseconds), whereas α4β2‐nAChR is more sensitive to ACh (EC_50_ is sub‐micromolar order) (Campling et al., 2013). In addition to this rapid electrophysiological response, nAChRs are also known to affect long‐term intracellular signalling through several intracellular signalling pathways (Akaike & Izumi, 2018; Schuller, 2009). The effects of nAChRs on intracellular signalling have been extensively studied as therapeutic targets for ischaemia, Alzheimer's disease and carcinomas (Akaike & Izumi, 2018; Kume & Takada‐Takatori, 2018; Li, Guan, et al., 2019). In particular, α7‐nAChRs stimulate cell proliferation via activation of PI3K/Akt and MAPK/ERK signalling (Akaike & Izumi, 2018; Kume & Takada‐Takatori, 2018; Larsen et al., 2019). In contrast, α4β2‐nAChR suppresses the signalling of PI3K/Akt and MAPK/Erk pathways. However, it is not clear whether α4β2‐nAChR directly affects these features (Akaike & Izumi, 2018; Kume & Takada‐Takatori, 2018; Larsen et al., 2019; Li, Guan, et al., 2019). Exploring the effects of α4β2‐nAChR on these signalling pathways may contribute to the clarification of the pathomechanisms of ADSHE and could indicate strategies for the development of treatments for focal epilepsy.

Nicotine intake upregulates the expression of several genes (Li et al., 2002), including Akt; however, acute and chronic administration of nicotine upregulate and downregulate phosphorylated ERK, respectively (Brunzell et al., 2003; Valjent et al., 2004). Before the ADSHE onset period (4 weeks of age), the expression of phosphorylated Akt and mCx43 in the thalamus and frontal cortex of S286L‐TG and wild type was comparable, whereas the expression of phosphorylated ERK was upregulated in S286L‐TG (Fukuyama & Okada, 2020). In contrast, after ADSHE onset (12 weeks of age), mCx43, phosphorylated Akt and ERK expression in S286L‐TG were upregulated compared to those in the wild type (Figure 2) (Fukuyama & Okada, 2020). A fundamental issue is establishing whether upregulated phosphorylated ERK prior to ADSHE onset contributes to epileptogenesis, or whether upregulated mCx43 and phosphorylated Akt play critical roles in ADSHE ictogenesis. It has been demonstrated previously that the propagation of epileptic discharges upregulates PI3K/Akt/mTOR signalling (Talos et al., 2018). Considering that the chronic administration of furosemide (MAPK/ERK inhibitor) prevents ADSHE onset, the combination of enhanced glutamatergic transmission and upregulated MAPK/ERK signalling in S286L‐TG shows that the congenital loss‐of‐function S284L‐mutant α4β2‐nAChR is critical for the development of epileptogenesis of S286L‐TG. In addition, the upregulation of both mCx43 and phosphorylated Akt induced by these two functional abnormalities is also relevant in primary and/or secondary ADSHE ictogenesis.

### 5.2 Possible pathogenesis of comorbid cognitive impairment

S284L‐TG rats do not show general behaviour and sensorimotor function abnormalities (Zhu et al., 2008), and other tests, such as the water maze, passive avoidance, forced swim and pre‐pulse inhibition tests, were also normal in this model. However, the spontaneous locomotor activity and social interaction test did detect a behavioural deficit in S284L‐TG (Zhu et al., 2008). These results suggest that S284L‐TG may develop autism‐like neuro‐cognition deficits (Zhu et al., 2008) and similar behavioural abnormalities were observed in V287L‐KI (Xu et al., 2011). These behavioural abnormalities of S284L‐TG and V287L‐KI associated with anxiety or social anxiety are insufficient and non‐specific, suggesting the acquisition of autism as a comorbidity of ADSHE. Therefore, more detailed behavioural analyses of S284L‐TG/S286L‐TG associated with autism should be performed, and it should be also clarified whether the behavioural abnormalities of S284L‐TG (autism‐like cognitive deficits or anxiety) are due to the loss‐of‐function mutant α4β2‐nAChRs.

Clinical evidence has emphasised that MDTN disturbance is a particularly relevant factor for cognitive dysfunction in psychosis, intellectual disability, autism and epileptic psychosis (Karlsen et al., 2014; Okada, Fukuyama, et al., 2020; Okada, Kawano, et al., 2020; Schuetze et al., 2016; Vertes et al., 2015). In particular, MDTN‐OFC glutamatergic transmission is considered critical in maintaining flexible stimulus–reward associations (Fukuyama et al., 2019; Pradel et al., 2018) due to the control of inputs from various cortical and subcortical regions (McCormick & Wang, 1991; Porrino et al., 1981; Russchen et al., 1987). Therefore, impairment of the controlling actions of MDTN‐OFC in S286L‐TG (Fukuyama et al., 2020b) probably contributes to the pathomechanisms of deficits of flexibility to environmental changes and communication with other individuals. Although exploring the pathomechanisms of ADSHE seizures has made steady progress, the pathomechanisms of cognitive impairment in ADSHE patients with insL‐ and S284L‐mutations remains to be clarified.

## 6 CONCLUSION

ADSHE rodent models have already shown robust results which were not available in single‐cell models, including that ADSHE‐mutant α4β2‐nAChRs contributes to the development of ADSHE epileptogenesis/ictogenesis by affecting various non‐cholinergic transmission systems, including tripartite synaptic transmission and neuronal networks. However, it should be noted that further efforts are needed to establish the validity and reliability of each rodent model. In particular, discrepancies in face validities between transgenic rats and KI mice models suggest that genetic backgrounds, general structure and dimensions of the relevant brain regions of rats are more appropriate for the development of ADSHE pathogenesis than those of mice. Several studies have shown that the seizure phenotype of the KI model is dependent on the ES cell type, indicating that the genetic background critically affects the quality and quantity of the seizure phenotype. Transgenic rat models have demonstrated more robust face validity, when compared with KI mice. However, the seizure pattern in S284L‐TG and S286L‐TG, showing three distinct ADSHE seizures during non‐REM sleep, was less severe than in most untreated ADNFLE patients. Additionally, the background of three ADSHE transgenic rat models was the Sprague–Dawley rat strain of the same colony. In contrast, experimental rats usually come from genetically diverse backgrounds, unlike inbred mice. If rat models with a homogeneous genetic background can be established, ADSHE rats produced using genome editing could become a breakthrough in elucidating the complicated interactions between responsible gene mutations and the background modifying factors in ADSHE pathogenesis.

Finally, we summarise the multistage age‐dependent and event (sleep/seizure)‐related pathomechanisms of ADSHE due to S284L‐mutation identified to date (Figures 1 and 2). Before ADSHE onset, the loss‐of‐function S284L‐mutant α4β2‐nAChR leads to relative GABAergic disinhibition and upregulated ERK signalling. During the critical period for ADSHE onset (4 to 8 weeks of age in S284L‐TG and S286L‐TG), upregulated ERK signalling partly increased mCx43 expression. The enhanced glutamatergic transmission induced by GABAergic disinhibition also moderately activates the function and expression of mCx43. The synergistic effect between GABAergic disinhibition and upregulated/activated mCx43 promotes epileptogenesis, generating interictal discharges, which accelerate seizure development by further upregulating/activating mCx43. In ADSHE epileptogenesis, the propagation of sleep spindle or pathological (i.e., interictal/ictal discharge) bursts drastically enhances the tripartite synaptic transmission associated with upregulated/activated mCx43. This activation results in the generation of ADSHE foci in the frontal cortex via hyperactivation of the thalamocortical pathway, or NPD, via hyperactivation of the thalamo‐subthalamic pathway. Once an ADSHE seizure leads to persistent activation of the mCx43 hemichannel, threshold reduction leads to seizure clustering.

### 6.1 Nomenclature of targets and ligands

Key protein targets and ligands in this article are hyperlinked to corresponding entries in the IUPHAR/BPS Guide to PHARMACOLOGY (http://www.guidetopharmacology.org) and are permanently archived in the Concise Guide to PHARMACOLOGY 2019/20 (Alexander, Christopoulos et al., 2019; Alexander, Fabbro et al., 2019; Alexander, Kelly et al., 2019; Alexander, Mathie et al., 2019; Harding et al., 2018).

## CONFLICT OF INTEREST

The author has no conflicts of interest to declare.
